# The Biological Role and Translational Implications of the Long Non-Coding RNA *GAS5* in Breast Cancer

**DOI:** 10.3390/cancers15133318

**Published:** 2023-06-23

**Authors:** Ilaria Grossi, Eleonora Marchina, Giuseppina De Petro, Alessandro Salvi

**Affiliations:** Division of Biology and Genetics, Department of Molecular and Translational Medicine, University of Brescia, 25123 Brescia, Italy; ilaria.grossi@unibs.it (I.G.); eleonora.marchina@unibs.it (E.M.); giuseppina.depetro@unibs.it (G.D.P.)

**Keywords:** *GAS5*, breast cancer, lncRNAs, ncRNAs

## Abstract

**Simple Summary:**

*GAS5* is a lncRNA that was identified decades ago as a key player in arresting the growth of mouse fibroblasts. *GAS5* may fold into an RNA structure that competes with the intracellular glucocorticoid receptor for binding to DNA targets located on the promoters of apoptosis-associated genes, thus controlling their expression. *GAS5* expression has been examined in a variety of human cancers, and its levels have been found to be lower in cancer tissues than in adjacent non-tumor tissues. In breast cancer (BC), *GAS5* has mainly been described as tumor suppressor lncRNA capable of promoting apoptosis and inhibiting cell proliferation. Low expression of *GAS5* is correlated with poor prognosis, and its expression is modulated by certain chemotherapeutic agents and during the acquisition of drug resistance. Due to its role and expression trend, new experimental approaches to augment or restore the cellular levels of *GAS5* may have important translational implications in BC as well as in other malignancies.

**Abstract:**

The lncRNA *GAS5* plays a significant role in tumorigenicity and progression of breast cancer (BC). In this review, we first summarize the role of *GAS5* in cell biology, focusing on its expression data in human normal tissues. We present data on *GAS5* expression in human BC tissues, highlighting its downregulation in all major BC classes. The main findings regarding the molecular mechanisms underlying *GAS5* dysregulation are discussed, including DNA hypermethylation of the CpG island located in the promoter region of the gene. We focused on the action of *GAS5* as a miRNA sponge, which is able to sequester microRNAs and modulate the expression levels of their mRNA targets, particularly those involved in cell invasion, apoptosis, and drug response. In the second part, we highlight the translational implications of *GAS5* in BC. We discuss the current knowledge on the role of *GAS5* as candidate prognostic factor, a responsive molecular therapeutic target, and a circulating biomarker in liquid biopsies with clinical importance in BC. The findings position *GAS5* as a promising druggable biomolecule and stimulate the development of strategies to restore its expression levels for novel therapeutic approaches that could benefit BC patients in the future.

## 1. Introduction

### 1.1. Breast Cancer

Breast cancer (BC) is the most common malignancy in women worldwide. It is a heterogeneous disease promoted by different risk factors, including the exposition to endogenous and exogenous estrogens, lifestyle, excessive food and alcohol consumption, obesity, and toxic environmental factors (pollutions, heavy metals, and chemicals) [1]. Approximately 15% of breast cancers are due to genetic predisposition and hereditary factors. Pathogenetic mutations in *BRCA1* and *BRCA2* (Breast Cancer Associated gene 1 and 2) genes have been extensively described in the literature [2,3], as well as other susceptibility genes with high or intermediate penetrance [4,5]. These genes are necessary for DNA repair, and their deactivation results in chromosomal instability that contributes to tumor transformation and carcinogenesis. The healthy mammary epithelium consists of the luminal cells (that produce milk) and basal cells that have contraction ability and favor milk secretion. BC is histologically classified into invasive ductal carcinoma (IDC), also known as invasive BC of no special type (NST), which accounts for 50% to 80% of all invasive cancers, and into “special subtypes” of BC. Special subtypes account for up to 25% of all BCs and include more than 17 distinct histological special types, with invasive lobular carcinoma (ILC) being the most common subtype. ILC accounts for approximately 10% of all invasive carcinomas [6,7]. BC is further classified based on the status of the intracellular estrogen receptor and progesterone receptor and the cell surface receptor HER2 (human epidermal growth factor receptor 2). On the basis of the expression profile, BC is further classified in five subtypes: (1) luminal A (approximately 50–60% of cases); (2) normal-like, which is a subtype of the luminal A group, but with worse prognosis (approximately 10% of the luminal A type); (3) luminal B (approximately 10% of cases); (4) HER2-enriched (approximately 20% of cases); and (5) basal-like triple-negative (TNBC) tumors (approximately 10% of cases). TNBC has a high risk of recurrence, with the worst prognosis among the different subtypes [8]. The curative options for BC are as follows: (a) surgical resection (75% of cases) of the tumor nodule/nodules to preserve the anatomy of the mammary gland when it is possible, or total removal (mastectomy); (b) radiotherapy used to destroy the remaining cancer cells after the surgery or to treat advanced BC (i.e., bone metastasis); (c) the use of hormone therapy for luminal BC with estrogen receptor antagonists in pre- and postmenopausal women; (d) targeted therapy such as the monoclonal antibody trastuzumab (Herceptin); and (e) treatment with inhibitors of CDK4/6 (cyclin-dependent kinase type 4 and 6) to inhibit cell cycle progression [9]. Following-up on patients with BC is essential for the best outcome, to monitor the response to therapies, and to verify the absence of relapse or metastasis in other organs such as the bones, lungs, liver, or brain.

### 1.2. Long Non-Coding RNAs

Long non-coding RNAs (lncRNAs) belong to the non-coding RNAs (ncRNAs) category. Based on their size, Mattick et al. recently suggested dividing the ncRNAs into three classes: (a) small RNAs less than 50 nucleotides in length (i.e., microRNAs and siRNAs); (b) ncRNAs generally 50–500 nucleotides in length, including RNA polymerase III (Pol III) transcripts such as tRNAs and 5S rRNA, small RNAs transcribed by RNA polymerase (Pol II) (i.e., small nuclear, snRNAs), and intron-derived small nucleolar RNAs (snoRNAs); (c) lncRNAs more than 500 nucleotides in length that are generally transcribed by Pol II [10]. GENCODE [11] and FANTOM consortium [12] identified ~20,000 and ~30,000 human lncRNA genes, respectively. More than 100,000 human lncRNAs transcripts have been annotated and catalogued in these databases. LncRNAs are transcribed by RNA polymerase I, Pol II, and Pol III, and are also processed from introns. With respect to protein-coding genes, lncRNAs can be classified into sense lncRNAs, antisense lncRNAs, bidirectional lncRNAs, intron lncRNAs, intergenic lncRNAs, and enhancer lncRNAs. The majority of lncRNAs are spliced and polyadenylated, but unlike mRNAs, they lack translational potential. While many lncRNAs are localized in the nucleus, a significant portion functions in cytoplasmic processes, and their expression is often cell and tissue-specific [13,14]. Numerous studies have described the involvement of lncRNAs in many physiological and pathological contexts. They participate in chromatin architecture and gene expression by regulating the transcription, RNA splicing, RNA stability, and protein translation [15]. LncRNAs play pivotal roles in numerous malignancies, and the aberrant expression of tumor-suppressive and/or oncogenic lncRNAs is frequently found in cancer cells. They are involved in carcinogenesis processes, such as proliferation, cell migration, invasion, epithelial–mesenchymal transition (EMT), and apoptosis [16,17].

### 1.3. GAS5

**Figure 1:**
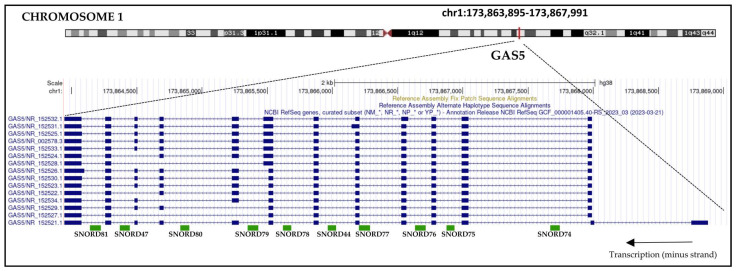
Human *GAS5* gene locus, structure, and transcript variants. *GAS5* is a lncRNA encoded by a sequence located at 1q25.1. In The Genome Browser database, 15 different RNA isoforms have been annotated with lengths of 510–725 nucleotides. *GAS5* transcript isoforms include up to 13 exons. *GAS5* is a gene harboring 10 small nucleolar RNA genes (snoRNA) in its introns (SNORD genes). (From Genome Browser; https://genome.ucsc.edu modified, accessed on 2 May 2023).

The growth arrest-specific 5 (*GAS5*) gene encodes for a lncRNA that was identified using a subtraction library approach to clone cDNA from growth-arrested NIH 3T3 mouse fibroblasts cells [18]. The human *GAS5* gene is located at 1q25.1, and it encodes for more than 20 different transcript variants with lengths of 510–725 nucleotides. The *GAS5* gene includes up to 13 exons (transcript variant NR_152521.1), as well as 10 small nucleolar RNA-coding sequences (snoRNA) in its introns (Figure 1).

**Figure 2:**
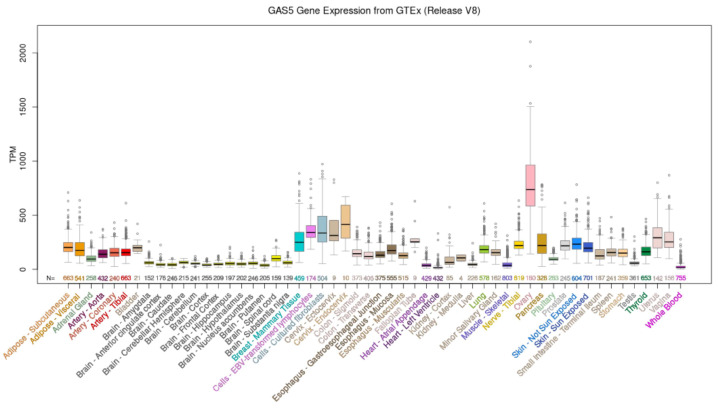
*GAS5* gene expression from GTEx (Release V8). The NIH Genotype–Tissue Expression (GTEx) project (https://commonfund.nih.gov/GTEx, accessed on 26 April 2023) was created to establish a sample and data resource for studies on gene expression in multiple human tissues. This track shows the median gene expression levels in 52 tissues and two cell lines (EBV-transformed lymphocytes and cultured fibroblasts) based on RNA-seq data from the GTEx final data release (V8, August 2019) obtained from UCSC genome browser (https://genome.ucsc.edu, accessed on 26 April 2023). This release is based on data from 17,382 tissue samples obtained from 948 adult post-mortem individuals.

The database LncBook 2.0 (integrating human long non-coding RNAs with multi-omics annotations) [19] provides conservation features of human lncRNA genes across 40 vertebrates. The conservation levels are expressed as Q50 = low conservation, Q75 = intermediate conservation, and Q90 = high conservation. According to the database, the level of *GAS5* conservation varies between Q50 and Q90 in apes (Q50 in *Gorilla gorilla gorilla*, Q75 in *Pan troglodytes*, and Q90 in *Nomascus leucogenys*). In rodents, *GAS5* shows Q50 conservation in *Mus musculus* and *Rattus norvegicus*, but Q90 in *Heterocephalus glaber*. In birds such as *Gallus gallus*, it exhibits Q50 conservation, while in the fish *Danio rerio* and the amphibian *Xenopus tropicalis*, it is not conserved. These data confirm an intermediate level of sequence conservation of *GAS5* across different species. Human *GAS5* is a well-studied lncRNA that widely contributes to various cell biology functions. *GAS5* acts as a decoy for a glucocorticoid receptor (GR) by mimicking the glucocorticoid response element (GRE) present on target DNA. The binding of *GAS5* with the GR blocks the binding between GR and GRE, inhibiting the transcription of genes modulated by GR that are involved in cell survival and apoptosis [20,21]. In addition, *GAS5* promotes osteoblast differentiation [22] and regulates the expression of insulin receptors in adipocytes [23]. According to the NIH Genotype–Tissue Expression (GTEx) project (https://commonfund.nih.gov/GTEx, accessed on 26 April 2023), *GAS5* is ubiquitously expressed in human tissues (Figure 2). Interestingly, high levels of *GAS5* expression were detected in female tissues and organs (breast, cervix, uterus, ovary), with the highest expression found in the ovary. This suggests its key role in the physiology and pathology of these anatomical districts.

## 2. *GAS5* Expression and Functions in BC

### 2.1. GAS5 Expression Is Dysregulated in BC Tissues

**Figure 3:**
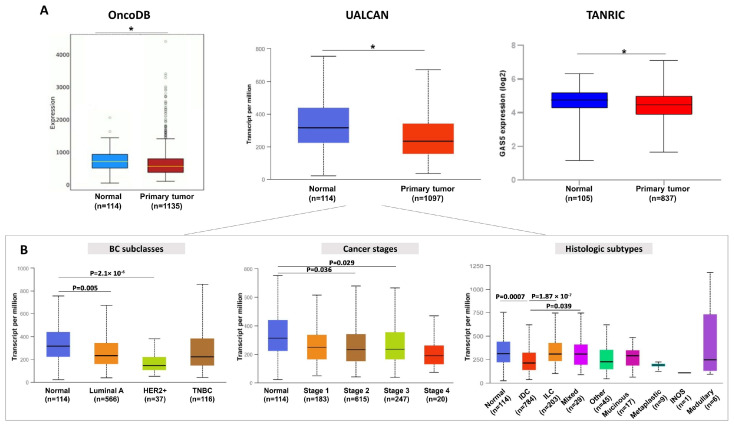
*GAS5* expression levels in TCGA datasets obtained by consulting different databases. (**A**) *GAS5* expression levels in primary BC tissues and normal tissues from TCGA datasets, evaluable in OncoDB, UALCAN, and TANRIC databases. (**B**) *GAS5* expression levels in the BRCA dataset from UALCAN based on BC subclasses, cancer stages, and histological subtypes. IDC, infiltrating ductal carcinoma; ILC, infiltrating lobular carcinoma; Mixed, mixed histology; INOS, infiltrating carcinoma not otherwise specified. * *p* < 0.05.

A growing number of studies have suggested that lncRNAs are abnormally expressed in human cancer. The expression patterns of certain lncRNAs, both in solid biopsies and biological fluids, are highly cancer-specific and can be strongly associated with clinicopathological characteristics, including the overall survival of cancer patients. All these features address the potential of lncRNAs as promising tools useful in cancer diagnosis, disease monitoring, and prognosis [24,25]. Concerning *GAS5*, several studies have reported that its expression is typically reduced in different cancer types, including hepatocellular carcinoma (HCC) [26,27], head and neck squamous cell carcinoma, glioblastoma, bladder, and non-small cell lung cancer (NSCLC) [28]. In BC, *GAS5* is generally downregulated in solid biopsies, as indicated by the bioinformatics analysis that we performed by consulting the OncoDB [29], UALCAN [30], and TANRIC [31] databases (Figure 3A). These publicly available online databases provide analyses of RNA-seq data from TCGA by considering the breast-invasive carcinoma (BRCA) dataset. The downregulation of GAS5 has been further observed among the major BC subclasses (Luminal A and HER2 positive) and in the predominant histological subtype (IDC, invasive ductal carcinoma) of the BRCA dataset (Figure 3B). Different published works have determined the *GAS5* levels in solid biopsies using qRT-PCR analysis and confirmed *GAS5* downregulation in tumor tissues from BC patients. Arshi et al. showed that *GAS5* expression in BCs is associated with the age of patients. *GAS5* levels were lower in the tissues of younger group (<45 years) compared with the older group (>45 years) [32]. Li et al. showed that *GAS5* levels were significantly decreased in HER2-positive BC specimens compared to corresponding non-tumor tissues (n = 20) [33]. Furthermore, low levels of *GAS5* were significantly associated with advanced TNM stages and grading, as well as poor overall survival (OS) and disease-free survival (DFS) [33,34]. Interestingly, *GAS5* expression was found to be positively correlated with unc-51 like autophagy activating kinase (*ULK*) *1/2* mRNA in BC clinical samples [35]. The over-expression of *GAS5* in BC cells up-regulated ULK1 and ULK2 protein expression, but not other autophagy-related proteins (Atgs), and led to autophagosome formation suggesting that the involvement of *GAS5* in autophagy should be further explored. Finally, *GAS5* was significantly downregulated in primary triple-negative BC (TNBC). In this BC type, the expression of *GAS5* was associated with tumor size, clinical stage, lymph node metastasis, and survival, suggesting that a high level of tissue *GAS5* could favor a good clinical outcome for BC patients [35,36,37]. Together, these results strongly support that the evaluation of *GAS5* expression in solid biopsies may have potential prognostic relevance in BC patients.

### 2.2. Different Mechanisms Are Involved in the Downregulation of GAS5 in BC

**Figure 4:**
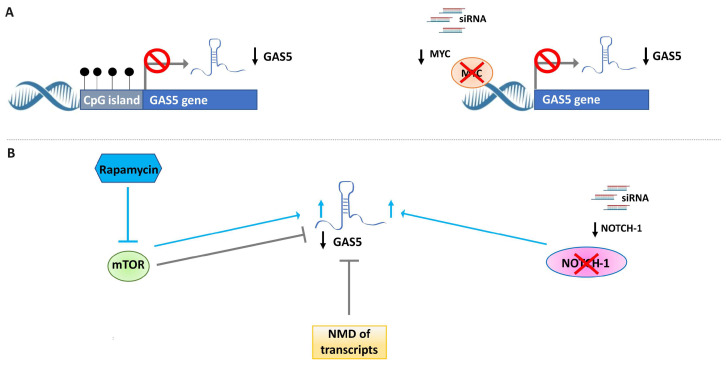
Schematic representation of mechanisms involved in the regulation of *GAS5* expression in BC. (**A**) *GAS5* expression can be downregulated by DNA methylation (left). MYC silencing (right) induced by siRNA molecules can decrease *GAS5* expression. Black circles indicate methylated cytosines. (**B**) Different pathways, including mTOR, NMD, and NOTCH-1, can be involved in the post-transcriptional regulation of *GAS5* in BC. Nonsense mediated decay, NMD.

As for other lncRNAs, the expression of *GAS5* can be regulated by different mechanisms at the transcriptional or post-transcriptional level (Figure 4). Among these, DNA methylation that occurs in the CpG islands located in the promoter region of the gene can affect the expression of the lncRNA. As indicated in UCSC Human Genome Browser (GRCh38/hg38; consulted in March 2023) [38], the *GAS5* gene is located on human chromosome 1q25.1 (chr1:173,858,997–173,867,989), and a CpG island has been identified upstream of the *GAS5* gene (chr1:173,868,035–173,868,779). Li et al. evaluated the DNA methylation of *GAS5* in TNBC tissues and cells using a methylation-specific PCR (MSP) assay [39]. They showed a significant increase in DNA methylation levels in TNBC tissues versus adjacent normal tissues. Regarding BC cell lines, they found that GAS5 was methylated and poorly expressed in TNBC cell lines (MDA-MB-231, MDA-MB-468, HCC1937, and MDA-MB-453), but partially demethylated and up-regulated in non-malignant human breast epithelial cells, MCF10A. Treating TNBC cells with the demethylating agent 5-Aza-2′-deoxycytidine (5-aza) was shown to determine the decrease in DNA methylation levels and the restoration of *GAS5* levels [39]. These data, also obtained in other cancers (such as melanoma [40] and cervical cancer [41]), support the finding that aberrant DNA methylation is one of the mechanisms involved in the downregulation of *GAS5*. At the post-transcriptional level, the expression of *GAS5* is modulated by the interplay between the mTOR and the nonsense-mediated decay (NMD) pathways. The mTOR pathway takes part in the regulation of 5′TOP-containing mRNAs through its downstream effectors (p70S6K and eIF4E/4E-BP1) [42]. The NMD pathway is involved in the degradation of transcripts containing stop codons in early exons. As reported in the literature, *GAS5* is considered a 5′-terminal oligopyrimidine tract (5′-TOP) gene [43], and is characterized by a short reading frame terminating with a stop codon within exon 3 (of 12) that is recognized by the NMD pathway. In actively growing cells, the activation of the mTOR signaling pathway regulates *GAS5* expression [44]. In support of this, the inhibition of mTOR activity by rapamycin determines the increase in cellular *GAS5* in different cell types, including BC cells [45,46]. Two additional well-known oncogenic pathways, Notch-1 and MYC, seem to be involved in the regulation of *GAS5* in BC cells. Pei et al. reported that the silencing of Notch-1 using siRNAs significantly increased the level of GAS5 in the T47D cell line [47]. Similarly, the lentiviral vector-mediated siRNA knockdown of MYC led to a decrease in *GAS5* expression levels in MCF7 cells [48]. Thus, *GAS5* expression may be regulated depending on the modulation of Notch-1 and MYC levels. Further in vitro and in vivo studies are required to elucidate the type of regulation, direct or indirect, exerted by Notch-1 and MYC on the expression of *GAS5* in BC.

### 2.3. GAS5 Functions as ceRNA of miRNAs in BC

The main biological function described in BC for *GAS5* is as competitive endogenous RNA (ceRNA). ceRNAs are ncRNAs which competitively bind microRNAs (miRNAs) and sequester miRNAs from their original target transcripts. Consequently, they avoid the degradation or expression inhibition of target transcripts induced by miRNAs at the post-transcriptional and translational levels. As in other cancers, *GAS5* can interact with different miRNAs and thus weaken the effect of miRNAs on mRNA targets. At the time of writing this review, we found five studies on BC reporting that *GAS5* acts as a ceRNA for four miRNAs with oncogenic functions (onco-miRs), including miR-21-5p, miR-221-3p, miR-196a-5p, and miR-378-5p [36,37,49,50], and the tumor-suppressor miR-216b [51]. miR-21-5p is the most commonly upregulated miRNA in solid and hematological malignancies and exerts its function by regulating tumor suppressor genes, like PTEN, TPM1 and PDC4. Zhang et al. verified the interaction between *GAS5* and miR-21-5p using RNA immunoprecipitation and RNA-pulldown assays [49]. Furthermore, in vitro experiments demonstrated the down and upregulation of *GAS5* after transfection with miR-21-5p mimics and anti-miR-21-5p, respectively. These data suggested the reciprocal regulation of miR-21-5p and GAS5 in BC cells. In triple-negative BC cells, *GAS5* has been shown to act as a ceRNA of miR-196a-5p, which targets FOXO1 [36]. The upregulation of *GAS5* in TNBC cells (i) increases FOXO1 expression, (ii) inhibits downstream PI3K/AKT activation, and (iii) decreases cell invasion. The over-expression of miR-196a-5p partially rescued the effects induced by the ectopic expression of *GAS5* in TNBC cells. In the same cell lines, Zheng et al. reported that *GAS5* promoted apoptosis by acting as a ceRNA of miR-378-5p which negatively regulated SUFU [37]. It is known that SUFU functions as a negative regulator of the Hedgehog signaling pathway and has a key role in promoting apoptosis. In BC cells resistant to adriamycin treatment, direct binding between *GAS5* and miR-221-3p was verified using a luciferase reporter assay and an RNA immunoprecipitation assay [50]. *GAS5* competed for miR-221-3p binding to regulate the expression of its target, dickkopf 2 (DKK2). The role of the GAS5/miR-221-3p/DKK2 axis in adriamycin resistance is explained in detail below. Finally, *GAS5* may also interact with miR-216b in BC cells, as suggested by the results of the dual-luciferase assay. In the same work, the authors showed that the inhibition of *GAS5* using siRNA determined (i) the decrease in the invasion ability of BC cells, and (ii) inhibition of E-cadherin, as well as the up-regulation of N-cadherin and Vimentin in cervical cancer cells (SiHa cells) [51]. These data suggest that *GAS5* may be involved in epithelial–mesenchymal transition (EMT) in cancer, and further studies will be fundamental in determining the role of *GAS5* in metastasis formation in BC.

In addition to its ceRNA role, *GAS5* has been recently described as mitochondria-associated lncRNA. Sang et al. reported that *GAS5* localizes in mitochondria, where it modulates tricarboxylic acid (TCA) flux by suppressing the formation of the metabolic enzymes complex formed by canonical members of the TCA cycle (fumarate hydratase, FH; malate dehydrogenase, MDH2; citrate synthase, CS) [34]. In BC cells, *GAS5* levels are decreased by glucose deprivation. A decreased availability of mitochondria-associated *GAS5* results in the formation of the CS-MDH2-FH complex, promoting the TCA cycle. These data indicate an important functional role for *GAS5* as a regulator of metabolism in BC cells.

## 3. *GAS5* as a Potential Biomarker and Therapeutic Target in BC

### 3.1. GAS5 Levels Modulate the Response to Apoptosis-Inducing Agents in BC Cells

In breast and other cancer cell lines, *GAS5* plays an important role in affecting cell growth and determining apoptosis [52]. As previously described, in BC cells, *GAS5* sponged miR-21 [49] and miR-196a-5p [36], determining the upregulation of the tumor suppressors PTEN, PDCD4, and FOXO1, which are key factors in the induction of apoptosis. By considering the close relationship between the activation of apoptosis and cancer therapy (i.e., radiotherapy and chemotherapy), the intracellular levels of *GAS5* may have important implications for cancer therapy responses due to its apoptosis-promoting activity. Consistent with this observation, Pickard et al. demonstrated that low *GAS5* levels attenuated the effects of chemotherapeutic agents (5-FU and docetaxel) and UV-C irradiation in terms of apoptosis induction, cell viability, and colony formation ability in in vitro BC models [46]. Particularly, reductions of 50–70% in *GAS5* levels in MCF7 and T-47D cells were strongly associated with attenuated cell death in response to treatment with 5-FU, docetaxel, or UV-C irradiation [46]. Conversely, MCF-7 cells transfected with the *GAS5*-overexpressing vector pCMVSPORT6 were more sensitive to UV irradiation and cisplatin-induced apoptosis [53]. However, it has been observed that, in the same cell line, the effect on apoptosis caused by TKI imatinib is independent of the cellular *GAS5* level. Taken together, these data suggest that *GAS5* is able to modulate the action of anti-cancer treatments in a selective way, likely due to the mechanisms of action of the different drugs. These findings could have important implications from a therapeutic point of view. Indeed, the increase in intracellular levels of GAS5 before the administration of chemo- or radio-based therapies may represent an innovative approach to improve the action of the treatments. Importantly, this goal could be achieved by acting on the mechanisms involved in *GAS5* regulation, such as DNA methylation and/or the mTOR pathway. As previously described, the demethylating agents, as well as the mTOR inhibitors, restore *GAS5* levels in BC cells. Therefore, functional studies should be addressed to prove the efficacy of innovative anti-cancer tools based on the combination of epigenetic drugs (i.e., the demethylating agent 5-aza) or mTOR inhibitors (i.e., rapalogues) with conventional treatments.

### 3.2. GAS5 Levels Are Modulated by Different Anticancer Drugs

*GAS5* expression levels are expected to impact the treatment response of BC cells. However, relevant studies have reported that different anti-cancer drugs alter the *GAS5* levels of BC cells. Among these, treatment with TKI sorafenib results in a significant increase in *GAS5* levels in HCC-1937 and MCF-7 cells. The authors hypothesized that the increased *GAS5* levels may be an indirect downstream effect of the sorafenib treatment [26]. Jiang et al. demonstrated that metformin, a lipophilic biguanide with discussed antitumor effects, increases *GAS5* levels in BC cells. The authors demonstrated that the levels of mTOR, phospho-m-TOR, and mitogen-activated kinase p-P70S6K proteins decreased after metformin treatment of breast cancer cells resistant to tamoxifen. The upregulation of *GAS5* resulted in growth inhibition of BC cells and increased apoptosis [54]. Similarly, natural compounds with antitumor properties have been found to modulate *GAS5* levels, including curcumin, a chemo-protective natural agent extracted from the rhizome of *Curcuma longa*. The exposure of three BC cell lines to dendrosomic-delivered curcumin significantly affected the increase in GAS expression as well as certain anti-cancer effects such as a decrease in cell viability, migration, and apoptosis induction. Interestingly, knocking down *GAS5* using siRNAs limited the effects on cell proliferation, apoptosis, and migration, suggesting that it might play a role in the anticancer action of curcumin in BC [55].

### 3.3. GAS5 Has Regulatory Functions in Drug Resistance of BC

Drug resistance remains a clinical challenge in the treatment of BC. An understanding of the mechanisms involved in the development of resistance is urgently needed to reduce the side effects of treatments and identify new therapeutic strategies for improving the outcomes of BC patients. Numerous studies have shown that lncRNAs can be considered important players in the regulation of drug resistance [56]. *GAS5* has been implicated in the activation of drug resistance through the regulation of different pathways. One of these pathways involves the membrane-associated protein ABCB1, a member of the superfamily of ATP-binding cassette (ABC) transporters that is responsible for decreased drug accumulation in multidrug-resistant cells and often mediates the development of anticancer drug resistance. Using RNA-seq technology, Chen et al. identified *GAS5* as one of the most downregulated lncRNAs in BC MCF7 cells resistant to adriamycin (ADR), an anthracycline widely used as a chemotherapeutic drug for BC. The over-expression of *GAS5* significantly increased the sensitivity of resistant cells to ADR by reducing cell proliferation capacity and increasing apoptosis [50]. Moreover, the upregulation of *GAS5* affected both the efflux function of ABCB1 and its expression. Mechanistically, the authors showed that *GAS5* acts as a ceRNA by competing for miR-221-3p binding to regulate its target DKK2, which is a critical receptor for activating the Wnt/β-catenin signaling pathway. Importantly, in vivo experiments have confirmed that the upregulation of *GAS5* reduces miR-221-3p and ABCB1 expression, increases DKK2 expression, and confirms the ability of *GAS5* to sensitize resistant cells to ADR. These important data suggest that GAS5 could revert ABCB1-mediated ADR resistance in ER-positive BC cells (MCF7) via the miR-221–3p/DKK2 axis by repressing the Wnt/β-catenin pathway. The role of lncRNA GAS5 in increasing tamoxifen sensitivity has been demonstrated in MCF-7 resistant cells and in vivo in nude mice. GAS5 may act as a ceRNA for miR-222, resulting in increased levels of the miRNA target PTEN. Knocking down miR-222 in tamoxifen-resistant MCF7 cells increased levels of *GAS5,* leading to higher sensitivity to the drug [57]. Similarly, *GAS5* overexpression also promoted sensitivity to ADR in triple-negative BC cells (MDA-MB-231 and MDA-MB-468) [39]. Approximately 13–15% of BCs are characterized by an amplification of ERBB2 that results in the activation of the HER2 pathway [1]. The epidermal growth factor 2 (ERBB2, formerly HER2 or HER2/neu) is a transmembrane receptor tyrosine kinase in the epidermal growth factor receptor family that correlates with a higher death rate in the absence of systemic therapy. Patients with HER2-positive BC benefit from HER2-targeted therapy, including anti-HER2 antibodies (such as trastuzumab) and TKIs (such as lapatinib) [58]. Li et al. identified *GAS5* as one of the most downregulated lncRNAs in HER2-overexpressing BC SKBR-3 cells made resistant to trastuzumab (SKBR-3/Tr). Since GAS5 functions as a ceRNA for miR-21, and miR-21 promotes tumor proliferation and invasion by targeting PTEN, the low levels of GAS5 in SKBR-3/Tr cells are related to low PTEN levels, favoring the aggressive properties of cancer cells. Interestingly, treatment with the TKI lapatinib suppressed the proliferation of SKBR-3/Tr cells by upregulating PTEN and GAS5 [33].

### 3.4. GAS5 May Sensitize BC Cells to Radiation Therapy

Radiation therapy is an integrative component of BC treatment [1]. In early BC, radiation therapy following surgery reduces locoregional recurrences and improves patient survival. In advanced BC, radiation therapy plays a crucial role in alleviating symptoms caused by metastasis, including those affecting the brain and bones [59]. However, cancer cells can become radioresistant, limiting the clinical benefit of this therapeutic approach. The role of *GAS5* in the radiation response shows promise. As mentioned above, *GAS5* was initially shown to impair the effects induced by UV-C irradiation, which was used as an in vitro apoptotic stimulus for BC cells [46,53]. More recently, Ma et al. reported that ionizing radiation inhibited *GAS5* expression in BC cells. Interestingly, the overexpression of *GAS5* in irradiated BC cells promoted apoptosis and significantly increased unrepaired DNA damage, which is one of the direct effects induced by ionizing radiation, leading to cell cycle arrest [60]. The authors also found that the upregulation of miR-21, known to be sponged by *GAS5*, reversed the effects of *GAS5* on the radiosensitivity of BC cells. These data suggest the potential role of *GAS5* in sensitizing BC cells to ionizing radiation through miR-21 and support further in vivo experiments to explore this function.

### 3.5. GAS5 Levels in Liquid Biopsies from BC Patients

Relevant evidence has indicated that the *GAS5* transcript is stable and detectable in biological fluids (i.e., plasma and serum). Many studies have evaluated the circulating levels of *GAS5* in plasma or serum from cancer patients and the promising results obtained in this field make circulating *GAS5* a potentially interesting non-invasive tool for cancer diagnosis, prognosis, and treatment response. In NSCLC and HCC, low levels of *GAS5* were found in plasma from cancer patients compared with healthy controls [26,61]. Interestingly, *GAS5* was also detected in exosomes isolated from the serum of NSCLC patients, displayed a significantly low level compared with healthy controls, and correlated with tumor size and TNM stage [62]. The circulating levels of *GAS5* in patients with solid cancers can be modulated in response to different anti-cancer therapies, including chemotherapy [63,64] and TKI [65]. Concerning BC, sparse but promising data have been published regarding the circulating levels of *GAS5*. Han et al. reported no significant differences in plasmatic levels of *GAS5* between BC patients and the healthy individuals. However, they found that *GAS5* plasma levels were significantly decreased at the postoperative stage compared with the preoperative stage in surgery-treated BC patients. A negative correlation was further shown between lymph node metastasis states and plasmatic *GAS5* levels after surgery [66]. Toraih et al. reported that *GAS5* was downregulated in the serum of BC patients compared with healthy subjects and non-cancer patients at risk of developing BC. Interestingly, lower *GAS5* levels in the serum of BC patients were related to metastasis and recurrence [67]. These data serve as a starting point for future research aiming to confirm the clinical significance of circulating *GAS5* as a biomarker for BC. The availability of larger cohorts of patients, as well as the use of standardized and sensitive protocols for lncRNA analysis (such as droplet-digital PCR [68]), should be considered to achieve this goal.

## 4. Conclusions

The lncRNA *GAS5* was discovered decades ago and has been predominantly studied in human cancer, particularly in breast cancer, where its downregulation is correlated with unfavorable clinical outcomes. Limited information is available on the role of human *GAS5* in cell biology and development, and to our knowledge, there is currently no characterization of its high expression in normal human female organs (breast, ovary, cervix and uterus) at a single-cell resolution in a spatial context. This lack of basic knowledge does not allow for a deep understanding of the role of *GAS5* in normal breast tissue and in BC. Nevertheless, altered expression of *GAS5* has been reported in diseases other than cancer, including neurogenerative disorders (i.e., Alzheimer’s [69]), autoimmune diseases (i.e., rheumatoid arthritis [70]), knee osteoarthritis [71], and preeclampsia [72]. Concerning BC, the extensive knowledge of *GAS5* expression in all subtypes of BC tissues highlights real translational implications of the data reported in the literature thus far. Given the advancements in RNA-based biotechnology, it is worth further exploring the translational potential of this promising RNA molecule. Several factors contribute to the importance of *GAS5* in breast cancer research: (i) *GAS5* downregulation is associated with an unfavorable clinical outcome of BC patients; (ii) altered *GAS5* levels have been detected following anti-cancer therapies, including chemotherapy and radiotherapy; and (iii) *GAS5* plays a role in sensitizing cancer cells to different types of treatment, promoting apoptosis, and in inhibiting the proliferation of cancer cells. As a tumor-suppressive transcript, therapeutic approaches aimed at augmenting *GAS5* expression in BC may be crucial in the development of novel drugs. However, it is important to address key challenges associated with ncRNA therapeutics, such as specificity, delivery, and tolerability [73].

## Data Availability

The data generated in this study can be found in the Genome Browser, (https://genome.ucsc.edu, accessed on 2 May 2023), OncoDB (https://oncodb.org/, accessed on 26 April 2023), UALCAN (https://ualcan.path.uab.edu, accessed on 26 April 2023), and Tanric https://bioinformatics.mdanderson.org/public-software/tanric/, accessed on 26 April 2023) databases.
